# How Should We Classify Kawasaki Disease?

**DOI:** 10.3389/fimmu.2018.02974

**Published:** 2018-12-14

**Authors:** Edoardo Marrani, Jane C. Burns, Rolando Cimaz

**Affiliations:** ^1^Rheumatology Unit, Department of Neurosciences, Psychology, Drug Research and Child Health, Meyer Children's Hospital, University of Florence, Florence, Italy; ^2^Department of Pediatrics, Kawasaki Disease Research Center, Rady Children's Hospital, University of California, San Diego, San Diego, CA, United States

**Keywords:** Kawasaki disease, etiopathogenesis, pediatric vasculitis, intravenous immune globulin (IVIg), coronary aneurysm

## Abstract

The exact classification of Kawasaki disease (KD) has been debated. Infectious disease specialists have claimed it as an infection with a classic immune responses to an as yet unidentified pathogen that localizes to the coronary arteries. Others have favored an autoreactive hypothesis that KD is triggered by an antigen that shares homology with structures in the vascular wall, and molecular mimicry resulting in an immune response directed to that tissue. Rheumatologists have classified it as a systemic vasculitis, while some immunologists have stressed the robust nature of the innate immune response that causes both systemic inflammation as well as damage to the coronary arterial wall and questioned whether KD falls within the spectrum of autoinflammatory diseases. This review will describe the evidences available up to now regarding these hypotheses.

Kawasaki Disease (KD) is an acute, self-limited vasculitis and is the most common cause of acquired heart disease in children of developed countries (1). The diagnosis of KD is clinical and it is based on a combination of fever and five clinical features (Figure 1).

**Figure 1:**
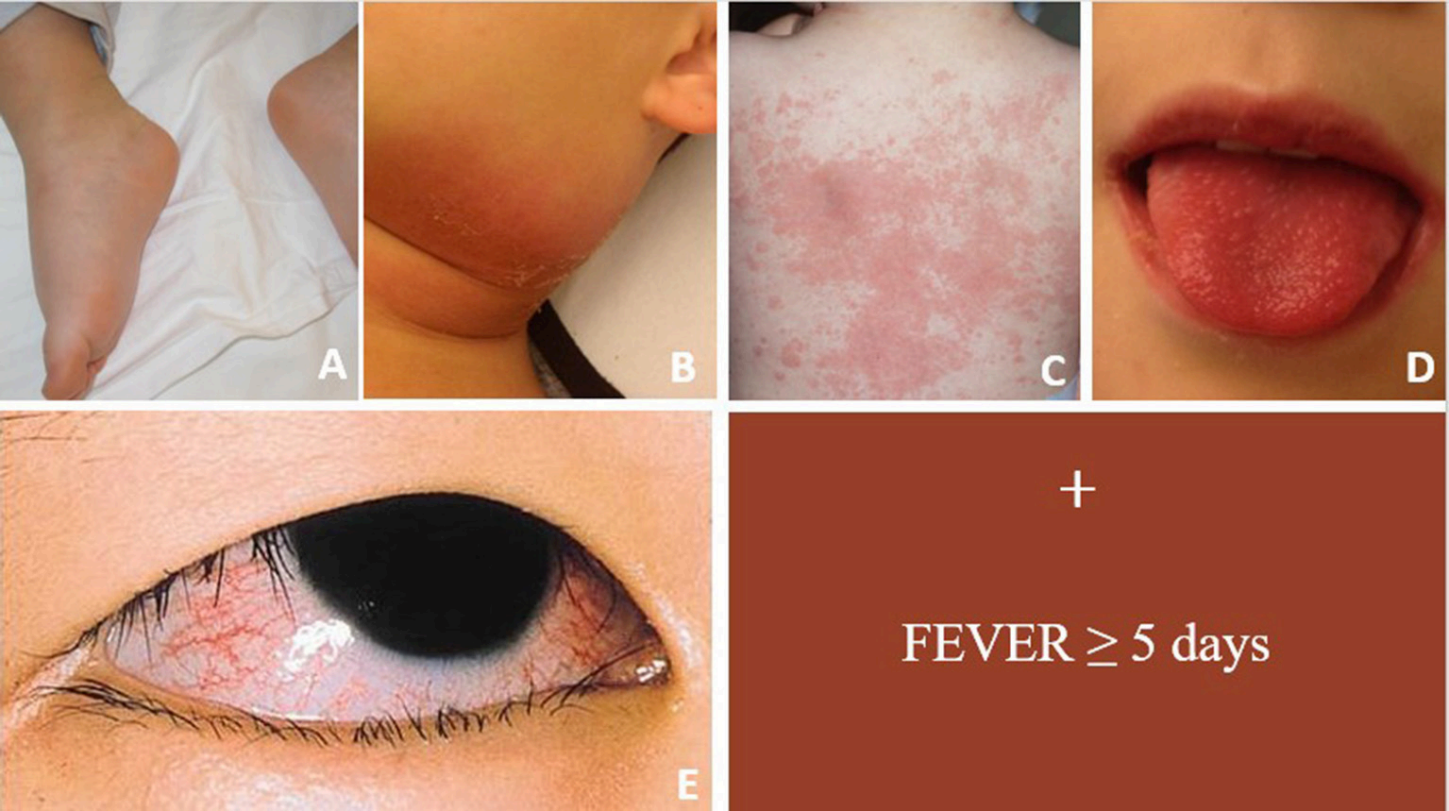
Figure showing main clinical signs of acute KD. For complete form ≥4 clinical criteria are required in conjunction with fever ≥5 days. **(A)** Erythema and edema of the hands and feet; **(B)** unilateral cervical lymphadenopathy; **(C)** polymorphous rash; **(D)** oropharyngeal changes; **(E)** bilateral bulbar conjunctival injection without exudate.

While in most cases the acute phase subsides within 2 weeks, even in untreated patients, up to 25% of all KD patients are at risk for significant coronary arteritis and subsequent development of coronary artery aneurysms (CAA) (2). Treatment with high dose intravenous immunoglobulin (IVIG) has been associated with a reduction of this risk to 3–6% and is recommended for all patients (3, 4).

A subset of patients with KD (10–20%) develops recrudescent or persistent fever at least 36 h after the end of the IVIG infusion (5). IVIG-resistant patients have a greater risk of cardiac complications. Furthermore, due to a limited knowledge of the exact mechanism of IVIG resistance, treatment of these patients remains challenging (6).

For decades, the exact classification of Kawasaki disease (KD) has been debated. Infectious disease specialists have claimed it as an infection with classic innate and adaptive immune responses to an as yet unidentified pathogen that localizes to the coronary arteries (7). Others have favored an autoreactive hypothesis that KD is triggered by an antigen that shares homology with structures in the vascular wall and the molecular mimicry results in an immune response directed to that tissue (1). Rheumatologists have classified it as a systemic vasculitis, thus lumping KD with other, usually chronic vasculitides of unknown etiology (8). Some immunologists have stressed the robust nature of the innate immune response that causes both systemic inflammation as well as damage to the coronary arterial wall and questioned whether KD falls within the spectrum of autoinflammatory diseases (9). Still others have labeled KD as autoimmune. Understanding the nature of the immune process that underlies KD will have direct implications for the direction that research initiatives should take and will influence how we attempt to modulate the immune system. The therapeutic approach for an autoimmune disease differs from an antigen-driven process, which, in turn, differs from a superantigen driven process. Therefore, we review here the evidence that supports different conclusions about the disease classification of KD.

## Evidence for Response to a Pathogen

The inflammation of the coronary arteries is the most striking feature of KD and it is responsible for the long-term sequelae, with children having giant aneurysms showing a relatively poor prognosis decades after the acute phase (10). The natural history of coronary damage in KD has been tracked by studies of tissues obtained at autopsy or at cardiac transplantation Thus, our knowledge of KD pathology is based on only the most severe cases and is therefore, incomplete. In the acute phase of fatal cases, a necrotizing arteritis is found in medium-sized vessels characterized by a neutrophilic and macrophage infiltration and destruction of the internal elastic lamina (11, 12). There is some evidence that this process may originate in the vasa vasorum. This innate immune response is followed by a subacute phase dominated by an adaptive immune response, in which infiltration of CD8+ cytotoxic T cells, plasma cells, and macrophages predominates. Myofibroblasts that may derive from endothelial or epithelial to mesenchymal transition can lead to luminal myofibroblast proliferation (LMP) (12). Progressive luminal obstruction by LMP with or without layering of thrombus along the aneurysmal wall are responsible for the long-term complications of KD, mainly coronary artery stenosis and acute coronary syndromes (13).

The myocardium is inflamed in all children with KD based on right endomyocardial biopsies during the acute disease (14, 15). In the convalescent phase, the myocardium may show diffuse, bridging fibrosis that may be related to low levels of persistent inflammation or chronic microvascular ischemia, as shown in pediatric patients (16) and in the long-term studies in adults (15).

Thus, the pathology of KD supports the concept of an early and robust innate immune response with infiltration of neutrophils into the arterial wall followed by an adaptive immune response with participation of T and B cells. Recently, Martin et al. studied the plasmablast expansion in KD. Plasmablasts are B cells (CD19+ CD20- CD27+ CD38+) transitioning to plasma cells that circulate in the peripheral blood cell compartment. KD patients showed a plasmablast response similar to children with infectious diseases, thus suggesting that circulating plasmablasts during KD produce antibodies that specifically target the agent responsible for the KD (17). Moreover, electron microscopy studies on bronchial epithelium of KD showed intracytoplasmic inclusion bodies (18) and oligoclonal IgA antibodies (19) suggesting a response to a pathogen entering through the upper respiratory tract. Rowley at al. synthesized artificial versions of these IgA antibodies and identified inclusion bodies suggestive of vesicles with virus-like particles (20).

However, gene expression profiling revealed that patients with acute KD have a unique gene expression pattern that is clearly divergent from patients with viral or bacterial diseases (21). In particular, Interferon type 1-stimulated gene expression was notably low in KD patients, when compared to patients infected with viral pathogens (22). Moreover, recently Wright et al. proposed a unique 13-transcript signature test as a tool to differentiate KD patients from those with bacterial, mycobacterial, and viral infections. This suggests that the host response of KD differs in important ways from the response of children infected with these other classes of agents (23).

## Evidence for an autoantibody or T Cell Driven Autoimmune Process

Autoimmunity is, by definition, a self-directed inflammatory response caused by dysfunction of mechanisms of tolerance (24). KD has been classified by some investigators, along with other vasculitides, as having an autoimmune origin (8). However, a considerable amount of evidence has progressively led to discrediting a role for autoimmunity in KD pathogenesis. First, the self-limited nature of KD and the low rate of recurrence argue against a primary autoimmune response, as autoimmune diseases typically manifest a chronic relapsing course. Furthermore, KD patients lack high autoimmunity burden or familial aggregation of autoimmune disease, as reported for other rheumatic diseases (25–27). National databases in high-prevalence populations from Japan and Korea have not reported any significant association with other autoimmune conditions, despite more than 20 years of observational studies (28, 29). Only a few studies have specifically addressed the association between KD and autoimmune diseases, such as autoimmune thyroiditis or celiac disease. Stagi et al. reported an increased prevalence of celiac disease in an Italian cohort of KD patients while the rate of autoimmune thyroiditis rate was similar between cases and controls (30). More recently, a cohort study conducted in a larger Brazilian population failed to show any association between celiac disease and KD (31), and only few case reports have been published regarding the co-occurrence of vitiligo in KD (32). Moreover, serological analysis has failed to demonstrate the consistent presence of disease-specific autoantibodies, with conflicting results regarding the presence of antinuclear antibodies (ANA), anticardiolipin (aCL), antineutrophil cytoplasmic antibodies (ANCA), and Anti-Human Cardiac Myosin Autoantibodies (33–37).

Therefore, it is unlikely that they play a major pathogenic role. Anti-endothelial cell antibodies (AECAs) have been more widely reported in KD (35, 36, 38, 39).

Although AECAs have been described in almost all primary systemic vasculitides, these autoantibodies are also present in several other diseases characterized by vascular involvement, including systemic lupus erythematosus, anti-phospholipid syndrome, rheumatoid arthritis, systemic sclerosis, and solid organ transplantation (40). So, it is not clear whether these are a primary driver for the inflammatory process or a secondary antibody response following endothelial cell destruction with the release of neo-antigens that could be the target of an antibody response. Moreover, an experimental model suggests that although AECAs from KD directly affect EC function *in vitro* and are associated with the development of some KD features, they are not able to initiate the vasculitic process or to induce coronary artery vasculitis (41).

Intriguingly, after IVIG administration, expansion of a natural regulatory T cell population (CD4+, FOXP3+, CD25^high^nTreg) is seen (42). The expansion of this population of nTreg requires presentation of the Fc region of IgG in a conventional MHC-restricted, TcR-mediated fashion by antigen presenting cells. These regulatory CD4+ cells may down-regulate the acute vasculitis by suppressing pro-inflammatory T cells in the lymph nodes and in secondary lymphoid organs and not directly in the inflamed coronary arteries. Thus, the resolution of the inflammatory process seems to be sustained both by secretion of IL-10 by tolerogenic myeloid dendritic cells (CD11c+ CD11b+ CD14+ CD4+ tmDCs) (43) and by the expansion of this population of Fc-specific nTreg cells (42).The selection of these Fc-specific Treg cells and their important role in immune regulation clearly differentiate KD from autoimmune diseases. Indeed, in children with a new onset autoimmune disease, failure to expand specific Treg populations may be associated with failure to abate the inflammatory process and consequently is responsible for the chronic course of these diseases. A defect in this Treg-mediated suppression of the pro-inflammatory T cell response has been documented only in KD patients with coronary artery abnormalities and in children with autoimmune disorders (42).

## Evidence for an Autoinflammatory Process

In 1999 McDermott et al. coined the term “autoinflammatory disease” (AIDs) to describe a group of rare diseases characterized by apparently unprovoked, recurrent systemic inflammation without any evident involvement of autoimmune pathways and no demonstrated causative infectious agents (44). AIDs are typically associated with aberrant activation of innate immunity due to genetic abnormalities in specific pathways of the inflammatory response (45). The “classical” monogenic AID are now known to include tumor necrosis factor (TNF) receptor–associated periodic fever syndrome, familial Mediterranean fever (FMF), hyperimmunoglobulinaemia D with periodic fever syndrome (HIDS), and cryopyrin-associated periodic syndromes (CAPS) (46). Irrespective of the specific underlying pathways, these syndromes are characterized by exaggerated Interleukin-1β (IL-1β) production. Active IL-1β initiates the inflammatory cascade and attracts monocytes and neutrophils causing tissue damage. This cytokine is also responsible for antigen-driven CD8+ T cell differentiation, proliferation, memory, and migration into tissues (45). Clinically, these syndromes are characterized by bouts of fever with a characteristic frequency and constellation of symptoms, including cutaneous rashes, lymphadenopathy, mucosal ulcerations, ocular findings, abdominal complaints, serositis, and musculoskeletal involvement (46). The overlap between the clinical presentation of KD and the prominent features of AIDs have suggested an autoinflammatory origin of KD, with activation of similar pathways that trigger innate immune responses.

The suggestion of an autoinflammatory origin of KD is further supported by gene expression data. Global gene expression profiling conducted on a large KD cohort by Hoang et al. (21) revealed that the overwhelming signature for acute KD involved signaling pathways of the innate immune system. Most importantly, activation of the IL-1 signaling pathway was identified as an essential driver of disease pathogenesis. Common features of the top 3 pathways for KD were the abundance of transcripts related to the NLRP3 inflammasome, IL-1α and IL-1β, and caspase 1. When compared to children with various infectious diseases, only patients with KD showed up-regulation of two key receptors IL1-receptor (IL1R) and IL-1 receptor accessory protein (IL1RAP). IL1R and IL1RAP are expressed on the cell surface, where IL1R directly binds to circulating IL1, while IL1RAP interacts with IL-1R for signal transduction and inflammatory cascade activation. Furthermore, studies have documented that IVIG treatment *in vitro* increases IL-1 receptor antagonist (IL-1Ra) expression in human monocytes and reduces *in vivo* IL-1β secretion by peripheral blood mononuclear cell in patients without CAA, when compared to KD patients developing CAA (47, 48).

In the *Lactobacillus casei* cell-wall extract (LCWE)-induced mouse model of KD (49), but not in the murine model induced by CAWS (water-soluble extracellular polysaccharide fraction obtained from the culture supernatant of *Candida albicans*) (50), IL-1β and IL-1α have been shown to induce myocarditis and aneurysm formation (51, 52). Even if the precise mechanism remains to be clarified, IL-1β signaling is likely to amplify intra-mural inflammatory processes, to prolong neutrophil survival and to enhance uncontrolled proliferation of luminal myofibroblasts. Moreover, CD8+ T cell infiltration of coronary artery wall and optimal effector function of pre-committed CD4+ T cells are also favored by IL-1β (53). The myocardial inflammation and aneurysm formation in the animal model were halted more effectively by treatment with IL-1 inhibitors compared to IVIG or TNF blocking agents (52). This experimental model has led the way to clinical use of IL-1 blocking agents in KD patients unresponsive to IVIG. At present, only few cases have been published (54–59) and the results from two clinical trials, currently ongoing in Europe and US, are awaited to elucidate the potential role of this therapeutic approach in KD (9).

However, several limitations to this “autoinflammatory hypothesis” for KD pathogenesis exist. As a first point, KD is not associated with alteration of single gene, as reported for AIDs, and genetic studies suggest a polygenic influence on disease susceptibility (60). Moreover, AIDs typically show a recurrent pattern, with a strict periodicity or with a variable interval between attacks. In KD, the disease is typically monophasic, even in absence of treatment, suggesting the activation of mechanisms of immune regulation that eventually halt the inflammatory response and induce immune memory.

## Summary: Classifying KD as an Immunizing Event

The available data as summarized above do not fit well with classic autoimmune or autoinflammatory diseases, as summarized in Table 1. The immune response is clearly influenced by genetic determinants that most likely shape the host response to one or more conventional antigens. However, the focused immunological assault on the coronary arteries remains a mystery. The rarity of KD in children under 6 months of age might be explained by the protective effect of maternal immunoglobulins and breast milk as there is a reduced incidence of KD in breastfed infants (61). Moreover, from epidemiologic studies we know that recurrent KD is rare and is influenced by host genetics with Japanese children having recurrence rates of 3–4%, while lower rates have been cited for children of European descent (62). Thus, the lack of recurrence seems to suggest that KD is usually an “immunizing” event associated with the development of immunological memory that protects the host from KD recurrences.

**Table 1:** Table representing the principal hypotheses for the etiopathogenesis of KD.

	**Response to a pathogen**	**Autoantibody or T cell driven autoimmune process**	**Autoinflammatory process**
Pros	• Histological features suggesting an early innate immune response, followed by an adaptive immunity • Plasmablast response such as in children with infectious diseases • Intracytoplasmic inclusions and oligoclonal IgA antibodies • Protective effect of breastfeeding • Low rate of recurrence • Seasonal variation and clusters of illness • Virtual absence of KD in adulthood	• AECAs antibodies in acute KD (conflicting results)	• Overlapping clinical features with AIDs • Increased expression of genes related to the NLRP3 inflammasome, IL-1a and IL-1b, and caspase 1 in blood • IL-1β and IL-1α implicated in myocarditis and aneurysm formation in LCWE -induced mouse model of KD • Efficacy of IL-1 blockade
Cons	• Unique gene expression pattern, differentiating KD from viral or bacterial diseases • No evidence for person-to-person transmission • Geospatial clustering at scales of 10-100 km	• Self-limited nature of KD • Low rate of recurrence • No concomitant autoimmune disease • No familial aggregation with other autoimmune disease • Selection of Fc-specific Treg cells in KD	• Polygenic nature of KD rather than a monogenic disease • Monophasic course

*For each hypothesis, data confirming or refuting are summarized. KD, Kawasaki disease; AIDs, autoinflammatory diseases; AECAs, anti-endothelial cell auto-antibodies; IL-1, interleukin 1; LCWE, Lactobacillus casei cell wall extract*.

Until the environmental trigger(s) is identified, the possibility of molecular mimicry, such as in rheumatic fever and streptococcal infection, cannot be excluded. If there is a genetically determined immunological defect in KD patients, it must be highly restricted to the response to a specific antigen since these children go on to be ostensibly immunologically normal with normal responses to vaccines and the panoply of childhood infectious diseases.

Genetic and immunological data from KD patients and data from the experimental mouse models of KD vasculitis have converged on the complex interplay between actors of both the innate and adaptive immune systems. However, as reported above, the exact mechanisms that lead to activation of the inflammatory cascade are still debated. According to available data, KD can be depicted as an acute inflammatory disease with genetic predisposition. The inter-ethnic differences in incidence rates, with a far higher incidence in Asian populations (63), the increased risk in first-degree relatives (64), and the increased severity of the disease in some populations support these findings. Epidemiological studies have found that KD shows seasonal variation and clusters of illness have been described (65, 66). Furthermore, the virtual absence of KD in adulthood suggests that the agent is widely distributed in the environment and thus genetically susceptible individuals will encounter the trigger for KD before they reach adulthood (7).

In conclusion, the weight of the evidence suggests that KD patients make an innate and adaptive immune response to one or more traditional antigens that confers lasting immunity against subsequent exposures in most patients. The autoreactive and autoimmune hypotheses lack evidence. The molecular mimicry hypothesis will be difficult to discard until the inciting antigens have been identified. The final classification of KD awaits identification of the inciting agent or agents.

## Author Contributions

All authors listed have made a substantial, direct and intellectual contribution to the work, and approved it for publication.

### Conflict of Interest Statement

The authors declare that the research was conducted in the absence of any commercial or financial relationships that could be construed as a potential conflict of interest.
